# Concurrent Loss of *PIGA* and *ZRSR2* in a Patient With Paroxysmal Nocturnal Hemoglobinuria and Myelodysplastic Neoplasm

**DOI:** 10.1111/ijlh.70198

**Published:** 2026-07-10

**Authors:** Fatma AlBulushi, Eric McGinnis

**Affiliations:** ^1^ Cytogenomics Laboratory, Department of Pathology and Laboratory Medicine Vancouver General Hospital Vancouver British Columbia Canada; ^2^ Department of Pathology and Laboratory Medicine, Faculty of Medicine University of British Columbia Vancouver British Columbia Canada

A 66‐year‐old man presented with persistent unexplained pancytopenia (hemoglobin 124 g/L, WBC 1.7 × 10^9^/L, platelets 97 × 10^9^/L). Peripheral blood immunophenotyping revealed a large PNH clone, with loss of CD157 and reduced FLAER uptake in 87% of neutrophils (Figure 1, Panel A) and 92% of monocytes (Figure 1, Panel B). 15% of RBCs demonstrated complete loss of CD59 (type III) and 0.2% showed partial loss (type II) (Figure 1, Panel C). Bone marrow examination was performed which showed adequate cellularity with borderline dysplastic changes and a normal karyotype. The patient exhibited a poor response to PNH‐directed therapy and worsening pancytopenia prompted repeat bone marrow evaluation which revealed a hypercellular marrow with marked granulocytic and megakaryocytic dysplasia, consistent with an underlying myelodysplastic neoplasm (MDS). Optical genome mapping (OGM), a genome‐wide cytogenomic technology increasingly implemented as a frontline test in the diagnostic evaluation of hematologic neoplasms, was performed as part of genomic profiling of the MDS clone. OGM identified a 636 kb deletion involving chromosome Xp22.2 resulting in loss of the *PIGA* gene (Figure 1, Panel D) in approximately 90% of cells, supporting the PNH clone previously identified by flow cytometry. The deletion also encompassed the *ZRSR2* gene which encodes a splicing factor recurrently mutated in myeloid neoplasms.

**FIGURE 1:**
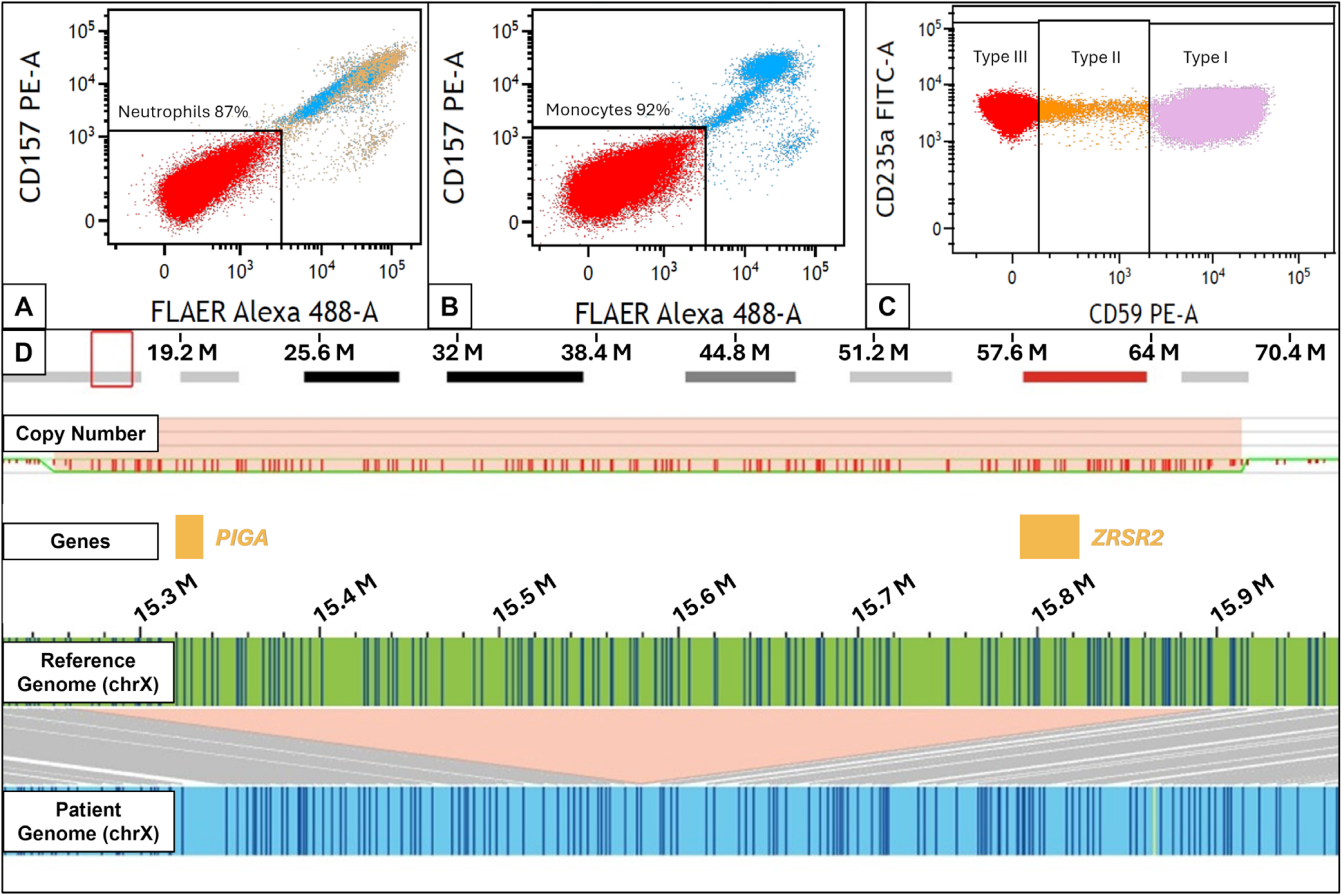
Flow cytometric and optical genome mapping findings in a patient with paroxysmal nocturnal hemoglobinuria (PNH) associated with myelodysplastic neoplasm (MDS). (A) Flow cytometric analysis of neutrophils demonstrating loss of CD157 expression and reduced FLAER binding in 87% of granulocytes (B) Flow cytometric analysis of monocytes showing loss of CD157 expression and reduced FLAER binding in 92% of monocytes (C) Flow cytometric analysis of red blood cells showing 15% of red cells with complete loss of CD59 expression representing type III PNH red blood cells and 0.2% of red cells with partial loss of CD59 expression representing type II PNH red blood cells. (D) Optical genome mapping demonstrating a 636 kb deletion (region highlighted in red) at chromosome Xp22.2 involving the *PIGA* and *ZRSR2* genes.

PNH results from somatic inactivation of the *PIGA* gene, leading to defective glycosylphosphatidylinositol (GPI) anchor biosynthesis and loss of multiple GPI‐anchored surface proteins, including CD55 and CD59 [1]. While most PNH‐associated *PIGA* alterations consist of point mutations or frameshift insertions/deletions, larger genomic deletions involving the *PIGA* locus are uncommon and may remain undetected by conventional diagnostic methods [2, 3]. In this case, OGM identified a 636 kb deletion at Xp22.2 encompassing *PIGA* resulting in protein loss of function. The estimated clone size by OGM seen in 90% of cells closely related to the proportions observed by flow cytometry in granulocytes and monocytes, highlighting the ability of OGM to provide both structural characterization and an assessment of clonal burden.

The patient subsequently developed worsening cytopenia, prompting repeat bone marrow examination that demonstrated dysplastic changes consistent with MDS. Notably, the deleted region at Xp22.2 also included the *ZRSR2* gene. Loss of function variants in *ZRSR2* may therefore have contributed to clonal evolution and disease progression in this patient. The concurrent deletion of both *PIGA* and *ZRSR2* suggests a single genomic event capable of driving both the PNH phenotype and MDS. This case highlights the value of high‐resolution genomic technologies, such as OGM, in identifying clinically significant cryptic deletions not routinely evaluated by technologies applied diagnostically in suspected myeloid neoplasms and refining the genomic characterization of hematological disorders beyond what is achievable with conventional cytogenetic approaches alone.

## Funding

The authors have nothing to report.

## Ethics Statement

The authors have nothing to report.

## Consent

The patient consented to the publication of the case.

## Conflicts of Interest

E.M. received in‐kind reagent supplies from Bionano and QIAGEN. E.M. serves as a consultant for, and has received research funding from, Jazz Pharmaceuticals. F.A. declares no conflicts of interest.

## Data Availability

The data that support the findings of this study are available from the corresponding author upon reasonable request.
